# *Sphingobacterium* sp. BHU-AV3 Induces Salt Tolerance in Tomato by Enhancing Antioxidant Activities and Energy Metabolism

**DOI:** 10.3389/fmicb.2020.00443

**Published:** 2020-04-03

**Authors:** Anukool Vaishnav, Jyoti Singh, Prachi Singh, Rahul Singh Rajput, Harikesh Bahadur Singh, Birinchi K. Sarma

**Affiliations:** ^1^Department of Mycology and Plant Pathology, Institute of Agricultural Sciences, Banaras Hindu University, Varanasi, India; ^2^Department of Botany, Institute of Science, Banaras Hindu University, Varanasi, India

**Keywords:** antioxidants, proteins, salt stress, *Sphingobacterium*, tomato

## Abstract

Salt tolerant bacteria can be helpful in improving a plant’s tolerance to salinity. Although plant–bacteria interactions in response to salt stress have been characterized, the precise molecular mechanisms by which bacterial inoculation alleviates salt stress in plants are still poorly explored. In the present study, we aimed to determine the role of a salt-tolerant plant growth-promoting rhizobacteria (PGPR) *Sphingobacterium* BHU-AV3 for improving salt tolerance in tomato through investigating the physiological responses of tomato roots and leaves under salinity stress. Tomato plants inoculated with BHU-AV3 and challenged with 200 mM NaCl exhibited less senescence, positively correlated with the maintenance of ion balance, lowered reactive oxygen species (ROS), and increased proline content compared to the non-inoculated plants. BHU-AV3-inoculated plant leaves were less affected by oxidative stress, as evident from a reduction in superoxide contents, cell death, and lipid peroxidation. The reduction in ROS level was associated with the increased antioxidant enzyme activities along with multiple-isoform expression [peroxidase (POD), polyphenol oxidase (PPO), and superoxide dismutase (SOD)] in plant roots. Additionally, BHU-AV3 inoculation induced the expression of proteins involved in (i) energy production [ATP synthase], (ii) carbohydrate metabolism (enolase), (iii) thiamine biosynthesis protein, (iv) translation protein (elongation factor 1 alpha), and the antioxidant defense system (catalase) in tomato roots. These findings have provided insight into the molecular mechanisms of bacteria-mediated alleviation of salt stress in plants. From the study, we can conclude that BHU-AV3 inoculation effectively induces antioxidant systems and energy metabolism in tomato roots, which leads to whole plant protection during salt stress through induced systemic tolerance.

## Introduction

Soil salinity is one of the major abiotic stresses that severely affect seed germination rate, plant growth, and productivity. Worldwide, around 20% of cultivated land and almost 50% of irrigated land is affected by salt (Machado and Serralheiro, 2017). Soil salinity restricts plant growth via osmotic and ionic stress. For instance, soluble salts present in the soil induce osmotic stress in roots, which hinders water acquisition in plant cells, and at high concentrations of salts, accumulation of sodium and chloride ions in plant cells cause ionic stress and can lead to nutrient deficiency. In addition, ionic stresses disturb the equilibrium of reactive oxygen species (ROS) in plant cells, which directly causes oxidative stress (Vaishnav et al., 2016a, b).

Salinity response in plants is a complex mechanism involving regulation of both physiological and molecular processes. The modification or activation of different metabolic processes during salt stress is controlled by the plant’s innate immunity and the habitat-imposed immunity provided by associated microbes (Vaishnav et al., 2019). A group of plant-beneficial microbes are known as plant growth-promoting rhizobacteria (PGPR), and they provide various benefits to the plants under both biotic and abiotic stresses. Application of salt-tolerant PGPR is a sustainable and cost-effective solution for cultivation in saline soil. Salt-tolerant PGPR can survive in saline soil and help plants tolerate salinity by several synergistic mechanisms, i.e., increasing osmolyte accumulation, phytohormone signaling, nutrient uptake, and antioxidant capacity (Sharma et al., 2016). A positive outcome of a plant’s interaction with beneficial microbes during salt stress is a promising way to improve crop productivity in saline soils. However, there is a need to understand the mechanisms of beneficial interaction between plant and microbes to alleviate stress.

How plant-associated microbes modulate host physiology to withstand stress conditions is a topic of interest. Recently, some attempts have been made to understand plant responses to salt stress with microbial inoculation, and these suggest the involvement of antioxidative machinery, osmolyte accumulation, and phytohormone signaling (Cao et al., 2017; Chanratana et al., 2019; Orozco-Mosqueda et al., 2019; Vimal et al., 2019; Yoo et al., 2019). However, targeting a single response and single plant tissue will miss the broader effect of plant–microbe interaction and also limit our understanding of stress signaling. In this context, advanced molecular tools and technologies will facilitate the characterization of plant–microbe interactions and may expand our understanding. Protein and gene transcript studies can provide meaningful insights for describing the interactions of plants with beneficial microbes under stress conditions (Cheng et al., 2012; Singh et al., 2017; Jaemsaeng et al., 2018; Kumar et al., 2019). Investigating and analyzing the plant’s root protein can provide a clear picture of the changes occurring at the time of microbe interaction. Only a few reports are available on combinational effects of beneficial microbes and salt stress through protein study, and these show that most of the expressed proteins in plants under salinity stress are related to transcription and translation factors, photosynthesis, lignin biosynthesis, and antioxidative and defense proteins (Cheng et al., 2012; Vaishnav et al., 2015; Singh et al., 2017). However, plant growth response to microbial application may vary with the experimental conditions, microbial diversity, and plant functional groups (Afroz et al., 2013).

Tomato is a vegetable crops grown all over the world for its nutritional value. Tomato is highly sensitive to salinity stress, which affects germination, vegetative growth, fruit set, development, ripening of fruit, and fruit quality. Previously, some attempts have been made to induce salt tolerance in tomato by inoculating other PGPR strains (Mayak et al., 2004; Tank and Saraf, 2010; Palaniyandi et al., 2014; Cordero et al., 2018). However, the molecular mechanism of PGPR-mediated salt tolerance in tomato plants is poorly explored. Therefore, the purpose of the present work is to (i) examine the plant growth-promoting properties of the strain *Sphingobacterium* sp. BHU-AV3; (ii) document the changes in tomato root proteins in response to salt stress when inoculated with the salt-tolerant strain BHU-AV3; (iii) compare the root and leaf tissues for contents of ions, proline, and different isoforms of antioxidative enzymes induced during salt stress upon inoculation of the strain BHU-AV3. This study extends our understanding of microbially mediated systemic tolerance in plants and motivates us to use microbial inoculants for the reclamation of salt lands.

## Materials and Methods

### Isolation, Identification, and NaCl Tolerance of the Bacterial Strain

BHU-AV3 was isolated from an agricultural field of Banaras Hindu University, Varanasi, India, on nutrient agar medium (NA) supplemented with 2% sodium chloride (NaCl). The molecular characterization of the BHU-AV3 bacterium was done by 16S rRNA gene sequencing using universal primers 27F (5′AGAGTTTGATCCTGGCTCAG 3′) and 1492R (3′ACGGCTACCTTGTTACGACTT 5′). The sequence was analyzed by Nucleotide BLAST (BLASTn) and further verified through the EzTaxon database. The salt tolerance ability of the BHU-AV3 bacterium was determined through inoculation in nutrient broth medium (NB) supplemented with 0.1–0.85 M NaCl and incubated at 28 ± 2°C.

### Determination of Plant Growth-Promoting (PGP) Activities

One-day-old bacterial culture (1.1 × 10^8^CFU) was used for the detection of PGP activities. Phosphate solubilization activity was determined by the modified method of Mehta and Nautiyal (2001). Bacterial culture was grown in NBRIP-BPB medium supplemented with phenol red dye (0.001%) and incubated at 28 ± 2°C for 3–4 days. The change in medium color from red to yellow indicated Pi solubilization.

Siderophore production was estimated on chrome azurol S agar (CAS) medium. BHU-AV3 culture was inoculated on CAS agar plates and incubated at 28 ± 2°C for 72 h. After incubation, the formation of orange halos around bacterial colonies represents a positive result for siderophore production (Schwyn and Neilands, 1987).

Indole-3-acetic acid (IAA) production by BHU-AV3 bacterium was determined as per the method of Gordon and Weber (1951). One-day-old bacterial culture was inoculated in NB medium containing Tryptophan (200 μg/mL) and incubated for 72 h with shaking (120 rpm) at 28 ± 2°C. Thereafter, complete culture was centrifuged and the supernatant collected. A volume of 1 mL of supernatant was mixed with 3 mL of Salkowski’s reagent (1 mL of 0.5 M FeCl_3_ in 50 mL of 35% HClO_4_) and kept in the dark for 30 min. The development of a pink color represents a positive result for IAA production.

### Plant Growth Assay With BHU-AV3 Inoculation

A loopful bacterial culture (24 h old) was dissolved aseptically in phosphate buffer saline (pH 7.4) and maintained at 10^8^ CFU mL^–1^. The bacterial cells were collected and resuspended in 1% of sterilized carboxymethyl cellulose (CMC) solution. Tomato seeds (cv. Kashi amrit) were surface-sterilized by 1.0% NaOCl for 1 min followed by 70% ethanol for 3 min and then rinsed with sterile distilled water three times. Sterilized seeds were dried on pre-sterilized blotting paper. The dried seeds were soaked into a bacterial suspension for priming, while only CMC-treated seeds served as a control. The primed seeds were kept in an incubator at 28 ± 2°C for 24 h. After incubation, seeds were sown in earthen pots filled with sterile soil. There were four treatment groups, i.e., (1) control, (2) salt (NaCl), (3) bacterial (BHU-AV3) inoculation, and (4) bacterial inoculation + salt. The complete experiment had a randomized block design, where three replications for each treatment were present under controlled conditions. Seven days after germination, salt treatment was given by irrigation with 50 mM NaCl for 4 days in the respective treatments. Seedlings were harvested at 21 days and evaluated for plant growth parameters.

### Evaluation of Physiological Responses of Plants to Salt Stress

Different physiological traits were measured to quantify the impact of salinity on tomato plants, as reported by Negrão et al. (2017).

Total free proline content and ion measurement were performed in root and leaf tissues. Proline content was measured according to the method of Bates et al. (1973). The chromophore-containing toluene was measured at 520 nm. The amount of proline was determined in μg/g fresh weight (FW) from a standard curve.

For Na^+^ and K^+^ ion measurement, samples were dried in a hot air oven at 60°C for 3–4 days and then ground into a fine powder. A 1-g dry powder sample was extracted with 5 ml of HNO_3_ at 37°C overnight. The filtered solution was diluted by distilled water and used for flame photometer analysis. Ion contents were measured in mg/g dry weight (DW).

Chlorophyll estimation was performed by the method of Moran and Porath (1980). The total chlorophyll content was quantified using the following formula, and the amount was expressed as μg Chlorophyll/g FW.

Chlorophyllcontent=[(ABS×6647.04)+(ABS×64720.27)]×5/sample⁢weight⁢(g)

The relative water content (RWC) was measured in plant leaves according to the protocol of Sade et al. (2015). Leaf samples were immediately placed in polythene bags after plucking to minimize water loss through transpiration. Samples were weighed to measure fresh weight (FW) and then kept in distilled water for 8 h. The leaf samples were placed between blotting papers to absorb excess water and then again weighed for turgid weight (TW). The samples were then oven-dried (60°C for 48 h) and again weighed to obtain dry weight (DW). The RWC was calculated by the following formula:

RWC(%)=(FW-DW)/(TW-DW)×100

### *In situ* Detection of ROS, Lipid Peroxidation, and Cell Death

*In situ* detection was estimated by the modified method of Ray et al. (2016). ROS production in the form of superoxide radicals was detected in tomato plant leaves. Leaves were kept in 25 mL of nitroblue tetrazolium (NBT) solution (10 μg/mL NBT dissolved in 50 mM phosphate buffer, pH 7.6) for 3 h in the dark. For lipid peroxidation, leaves were stained with Schiff’s reagent for 1 h, and aldehyde formation was detected as the end product of lipid peroxidation. Cell death was analyzed by immersing leaves in 0.1% Evan’s blue solution for 15 min. After staining, leaves were boiled in 95% (v/v) ethanol for 30 min in a water bath. Thereafter, leaves were kept in 40% glycerol before examination.

### Antioxidant Enzyme Activity Assays and Zymography

Antioxidant enzyme activities were performed in root and leaf tissues of tomato plant. The crude protein was extracted according to the method of Qureshi et al. (2013). One gram of tissue was homogenized in 4 mL of 100 mM potassium phosphate buffer (pH 7.4) containing 1 mM ethylenediaminetetraacetate (EDTA), 2% polyvinylpyrrolidone (PVP), and 1 mM phenylmethylsulfonyl fluoride (PMSF). The crude extract was centrifuged at 15,000 × *g* for 20 min at 4°C, and the supernatant was then collected; this was used as an enzyme extract. Total protein content was estimated according to the Bradford (1976) method.

Determination of peroxidase (POD) enzyme activity and zymography of its isoforms were performed as per the method of Kumari et al. (2015). POD activity was expressed in U/mg protein. POD isoforms were identified by native-polyacrylamide gel electrophoresis (PAGE) on 10% acrylamide gel. Polyphenol oxidase (PPO) activity was quantified as per the method of Weisany et al. (2012), and activity was expressed in U/mg protein. PPO isoforms were identified according to the method of Ramamoorthy et al. (2002). The native-PAGE gel was immersed in 0.1% p-phenylene diamine for 30 min. Thereafter, the solution was discarded and the gel was exposed to 20 mM catechol.

Superoxide dismutase (SOD) activity was determined according to the method of Qureshi et al. (2013), in which photo-reduction of nitroblue tetrazolium chloride (NBT) was measured at 560 nm. A 50% photo-reduction of the NBT was measured as one unit of SOD enzyme, and the activity was expressed in U/mg protein. Zymography of SOD isoforms was performed as per the method of Kumari et al. (2015).

### Extraction of Total Proteins and 1D SDS-PAGE, Trypsin Digestion, and In-Gel Extraction of Peptides for MALDI

Total proteins were extracted from root tissue by the phenol extraction method (Isaacson et al., 2006). The soluble fraction of extracted proteins was subjected to separation on 10% polyacrylamide gel using SDS-PAGE (Laemmli, 1970). The differentially expressed protein bands in bacteria-inoculated salt-stressed plants (T4) were observed and selected for trypsin digestion. Bands were excised from the gel and further processed for trypsin digestion as per the method of Mahajan et al. (2014). Peptides were extracted and submitted for peptide mass fingerprint (PMF) using a MALDI-TOF mass spectrometer. The generated data were searched for on the Swiss-Prot database using the MASCOT search engine (Matrix Science, London, United Kingdom).

### Expression Analysis of the m-RNA Genes of Selected Proteins Using qRT PCR

Root tissues were crushed in liquid nitrogen immediately after harvesting, and RNA was extracted with Trizol (Merck GeNei). The extracted RNA was treated with DNase, which was further used for cDNA synthesis. RNA was reverse transcribed by using Avian Myeloblastosis Virus (AMV) reverse transcriptase and Oligo (dT)_18_ primer. qRT–PCR was performed in a real-time PCR system (Bio-Rad Laboratories) using Eva Green SYBER Master Mix. The primers of selected genes were designed through IDT software; details are given in Table 1. The delta–delta CT method was applied to compare relative expression. The actin gene was chosen for normalization.

**TABLE 1:** Details of primers used in the qRT-PCR study.

**Gene name and accession number**	**Forward primer**	**Reverse primer**
Catalase (*CAT*) (NM_001247898)	TCGCGATGGTGCTATGAACA	TGTCTTGCCTGTCAGGTTCC
Enolase *(PGH1*) (NM_001247151)	GGCAGGTTGGGGTGTAATGA	CAATCTCAACACTTGGAACTGC
ATP synthase (KY887588)	GGTGAACGTACTCGGGAAGG	TGCTTGGACGAAACGGAAGA
Thiamine (*ThiC*) (NM_001317405)	CTTTCCGGGGATGAACCACA	ATTGGCTCCAACTCAGGGTG
Elongation factor-1α (*EF-1*α) (XM_004240531)	GTGCATTTGATGAGCACGGA	AGCAGTGACCAAGACTGTGT
Actin (NM_001330119)	TGGCTCCTAGCAGCATGAAG	ACACTACAATTGCATCTCTGGTC

### Statistical Analysis

The data obtained were subjected for significance analysis through analysis of variance (ANOVA) testing. Then, *post hoc* testing was performed using the DMRT test. All analysis described was performed in SPSS software version 11.5.

## Results

### Molecular Characterization and PGP Attributes of BHU-AV3 Isolate

**FIGURE 1:**
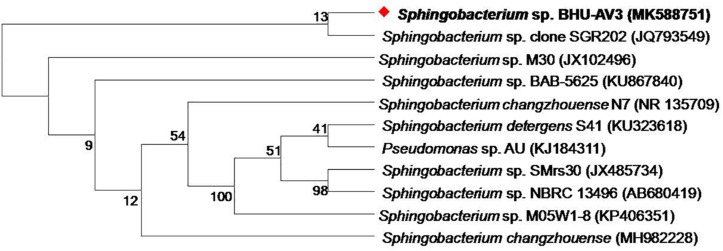
Phylogenetic tree constructed by using neighbor-joining analysis between BHU-AV3 isolate and reference bacterial sequences retrieved from GenBank based on 16S rDNA sequences. The tree shows the phylogenetic position of BHU-AV3 isolate among the genus of *Sphingobacterium*. GenBank accession numbers are given in parentheses. Numbers at nodes indicate percentages of bootstrap support, based on 1,000 resample datasets. Evolutionary analyses were conducted in MEGA7.

**FIGURE 2:**
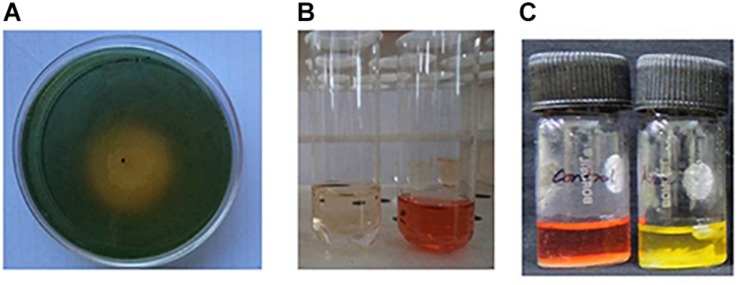
Qualitative estimation of plant growth-promoting properties of BHU-AV3 isolate. **(A)** Siderophore assay – yellow zone indicates positive result; **(B)** IAA production assay – development of pink color indicates positive result; **(C)** Pi solubilization test – development of red to yellow color indicates positive result.

As part of a program to discover salt-tolerant PGPR, BHU-AV3 isolate was recovered from an agricultural field. The isolate is most closely related to *Sphingobacterium*, with 99% identity based on 16S rDNA gene sequence analysis. The sequence data of BHU-AV3 has been deposited to the GenBank database with the accession number MK588751 (Figure 1). Screening of the PGP traits of strain BHU-AV3 revealed characteristics of phosphate solubilization, siderophore production, and indole-3-acetic acid (IAA) production (Figure 2). The strain BHU-AV3 is moderately halophilic, as it tolerates NaCl up to 4% (w/v).

### Effect of BHU-AV3 on Plant Growth Parameters Under Salt Stress

**FIGURE 3:**
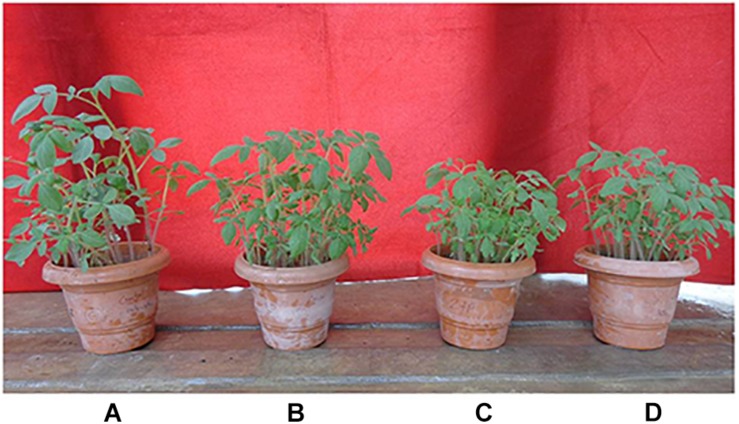
Effect of bacterial (BHU-AV3) inoculation on tomato plant growth under salt stress. **(A)** Control plants; **(B)** bacteria (BHU-AV3)-inoculated plants; **(C)** salt (NaCl) treatment; **(D)** bacterial inoculation + salt.

Salt stress caused a significant negative effect on plant growth parameters. All growth parameters were reduced under salt stress as compared to the control condition. However, BHU-AV3 was able to protect plants from severe damage due to salt toxicity (Figure 3). BHU-AV3-inoculated plants (T4) registered significantly enhanced shoot and root length (44 and 51.3%) as compared to T2 treatment. Similarly, bacterial inoculation significantly enhanced plant biomass as compared to un-inoculated plants under salt stress. T4 treatment showed an increase of 54% biomass compared to the un-inoculated control plants (T2) under salt stress (Table 2). Under non-salt conditions (T1 and T3), both treatments showed similar trends in plant growth parameters.

**TABLE 2:** Effect of bacterial (BHU-AV3) inoculation on tomato plant growth parameters under salt stress conditions.

**Treatments**	**Shoot length (cm)**	**Root length (cm)**	**RWC (%)**	**Chlorophyll (μg/g FW)**	**Biomass content (g)**
T1	18.6 ± 1.2^a^	6.7 ± 0.8^a^	85.6 ± 3.7^a^	80.9 ± 4.3^a^	0.28 ± 0.03^a^
T2	9.0 ± 0.6^c^	3.4 ± 0.4^c^	34.0 ± 2.6^c^	45.0 ± 3.2^c^	0.13 ± 0.04^c^
T3	16.5 ± 0.9^a^	7.8 ± 0.6^a^	88.6 ± 3.1^a^	84.6 ± 2.9^a^	0.31 ± 0.03^a^
T4	13.0 ± 0.7^b^	5.2 ± 0.7^b^	65.0 ± 2.9^b^	70.0 ± 2.0^b^	0.20 ± 0.02^b^

**T1, control; T2, salt (NaCl); T3, bacterial (BHU-AV3) inoculation; and T4, bacterial inoculation + salt. Values represent the mean ± SD, n = 3. Different superscript letters in the same column are significantly different (p = 0.05, DMRT analysis was carried out).**

### Effect of BHU-AV3 on Physiological Response to Salt Stress

A significant decrease in chlorophyll content was observed under salt stress. However, the BHU-AV3-inoculated plants (T4) had significantly higher (56%) chlorophyll content compared to un-inoculated plants (T2). The relative water content in tomato plants was remarkably reduced under saline conditions. Nevertheless, T4-treatment plants accumulated more water content (91%) compared to T2-treatment plants (Table 2). In addition, in terms of ion contents, Na^+^ content was significantly increased under salt stress conditions. In T2 treatment, plant roots had 190% higher Na^+^ content with respect to non-salt treatment (T1), whereas BHU-AV3-inoculated plant root (T4) had only 125% higher Na^+^ content than non-salt treatment (T3). On the other hand, the K^+^ content was significantly decreased in both salt treatments (T2 and T4). However, in T4 treatment, the plants exhibited a smaller decrease in K^+^ content (29%) compared to T2 plants (50%) from their respective controls. Hence, a 5-fold increment in Na^+^/K^+^ was recorded in T2 plant roots, whereas only a 2.2-fold increment was found in T4 plant roots compared to T1- and T3-treatment plants, respectively. T4-treatment plant leaves showed significantly less accumulation of Na^+^ content (36%) as compared to T2-treatment plant leaves (Table 3). Proline content was increased in both salt stress treatments (T2 and T4). Remarkably and in contrast to T2 treatment, T4-treatment plant roots had an increment (40%) in proline content. However, leaf tissue accumulated less proline content in T4 (27%) as compared to T2 plants (Table 3). These results suggest that strain BHU-AV3 relieved the negative effect of Na^+^ ion toxicity on tomato plant physiology under salt stress. All of these physiological parameters were observed at constant levels in non-salt stress treatments (T1 and T3).

**TABLE 3:** Effect of bacterial (BHU-AV3) inoculation on ion and proline content in different tomato plant tissues under salt stress conditions.

**Treatments**	**Na^+^ (mg/g DW)**		**K^+^ (mg/g DW)**		**Na^+^/K^+^**		**Proline (μg/g FW)**	
	**Root**	**Leaf**	**Root**	**Leaf**	**Root**	**Leaf**	**Root**	**Leaf**
T1	6.1 ± 0.7^c^	3.3 ± 0.4^c^	10.7 ± 0.5^a^	8.7 ± 0.6^a^	0.5 ± 0.2^c^	0.3 ± 0.01^c^	87.0 ± 3.2^c^	45.0 ± 2.6^c^
T2	17.7 ± 1.2^a^	11.8 ± 0.8^a^	5.0 ± 0.3^c^	3.2 ± 0.7^c^	3.5 ± 0.6^a^	3.6 ± 0.8^a^	131.0 ± 4.3^b^	114.0 ± 4.1^a^
T3	5.8 ± 0.6^c^	3.6 ± 0.6^c^	11.5 ± 1.0^a^	9.5 ± 0.5^a^	0.5 ± 0.2^c^	0.3 ± 0.01^c^	83.0 ± 2.4^c^	42.0 ± 3.1^c^
T4	13.1 ± 1.9^b^	7.6 ± 1.2^b^	8.1 ± 0.6b	7.0 ± 0.9b	1.6 ± 0.5^b^	1.0 ±0.2b	184.0 ± 3.5^a^	83.0 ±5.2b

**T1, control; T2, salt (NaCl); T3, bacterial (BHU-AV3) inoculation; and T4, bacterial inoculation + salt. Values represent the mean ± SD, n = 3. Different superscript letters in the same column are significantly different (p = 0.05, DMRT analysis was carried out).**

### Effect of BHU-AV3 on Antioxidant Enzyme Activity in Response to Salt Stress

**FIGURE 4:**
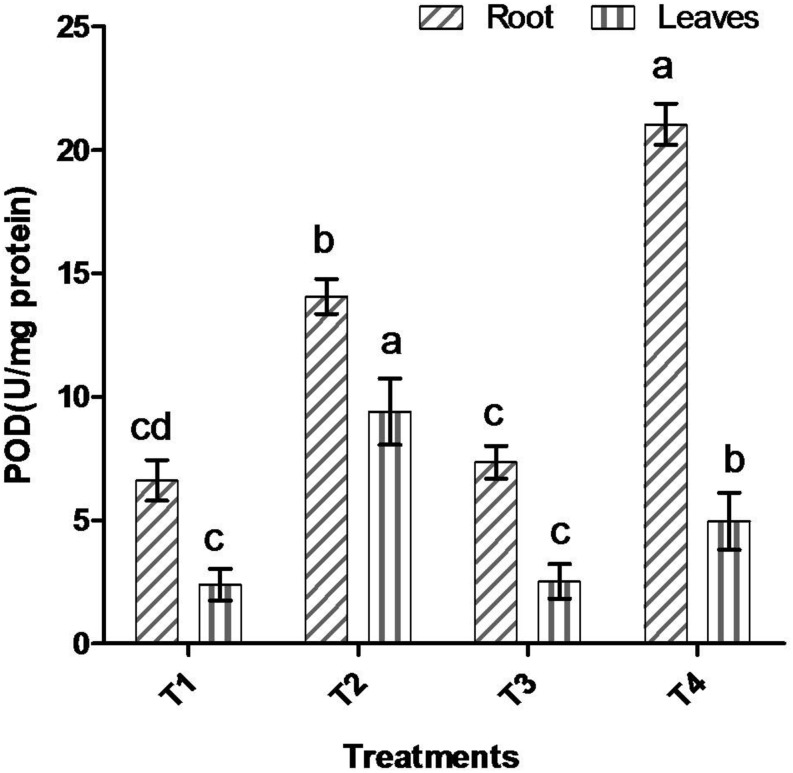
Effect of bacterial (BHU-AV3) inoculation on peroxidase (POD) activity in root and leaves under salt stress. T1 – control, T2 – salt (NaCl), T3 – bacterial (BHU-AV3) inoculation, and T4 – bacterial inoculation + salt. Values represent the mean ± SD, *n* = 3. Different letters on each bar indicate significant differences (*P* = 0.05) after DMRT test.

**FIGURE 5:**
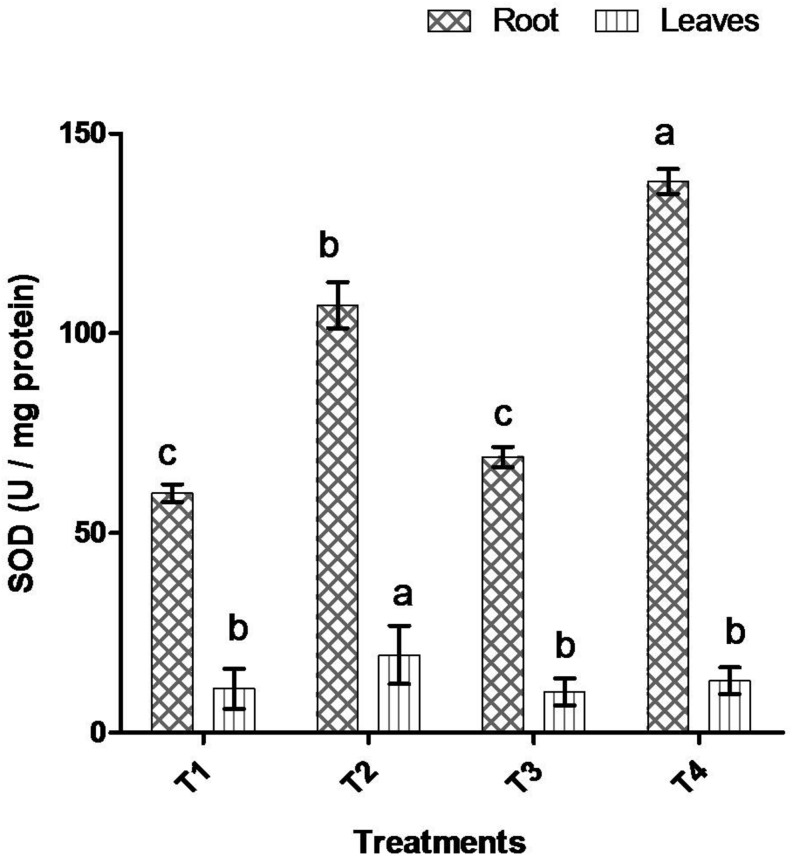
Effect of bacterial (BHU-AV3) inoculation on superoxide (SOD) activity in root and leaves under salt stress. T1 – control, T2 – salt (NaCl), T3 – bacterial (BHU-AV3) inoculation, and T4 – bacterial inoculation + salt. Values represent the mean ± SD, *n* = 3. Different letters on each bar indicate significant differences (*P* = 0.05) after DMRT test.

**FIGURE 6:**
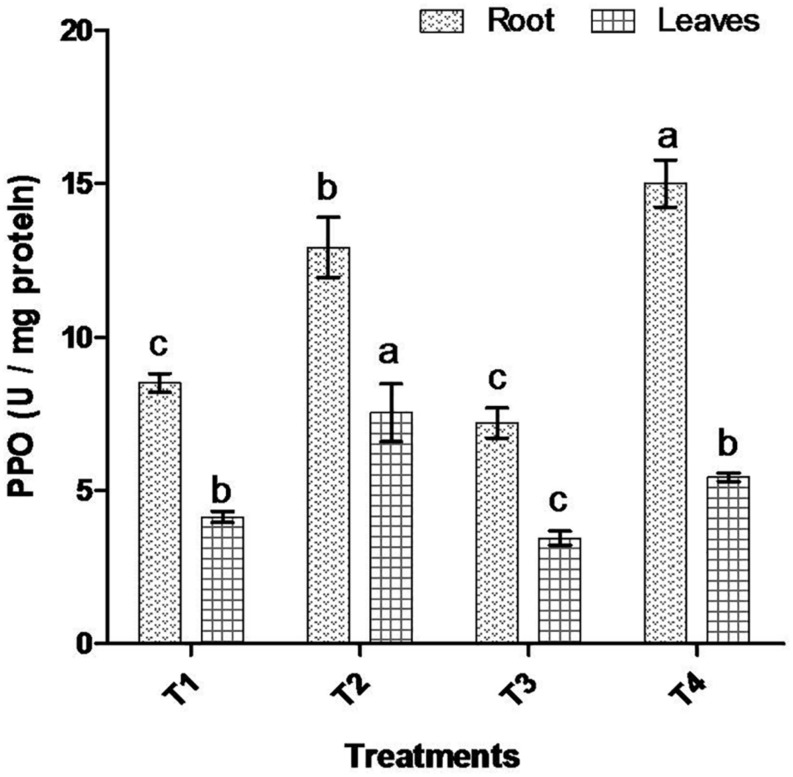
Effect of bacterial (BHU-AV3) inoculation on dismutase polyphenol oxidase (PPO) activity in root and leaves under salt stress. T1 – control, T2 – salt (NaCl), T3 – bacterial (BHU-AV3) inoculation, and T4 – bacterial inoculation + salt. Values represent the mean ± SD, *n* = 3. Different letters on each bar indicate significant differences (*P* = 0.05) after DMRT test.

**FIGURE 7:**
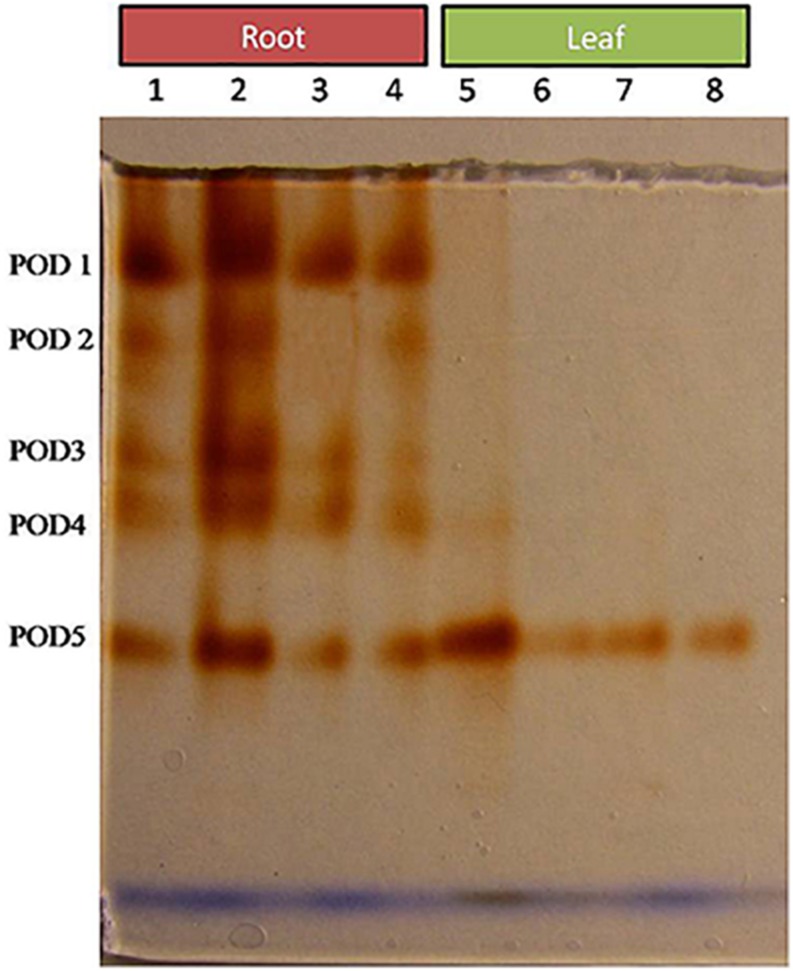
Zymography of POD isoforms expressed in root and leaves upon bacterial (BHU-AV3) inoculation under salt stress. Lanes 1 and 5 – salt treatment; Lanes 2 and 6 – bacterial (BHU-AV3) inoculation + salt; Lanes 3 and 7 – bacterial inoculation; Lanes 5 and 8 – Control. POD 1–5 represents number of isoforms expressed.

**FIGURE 8:**
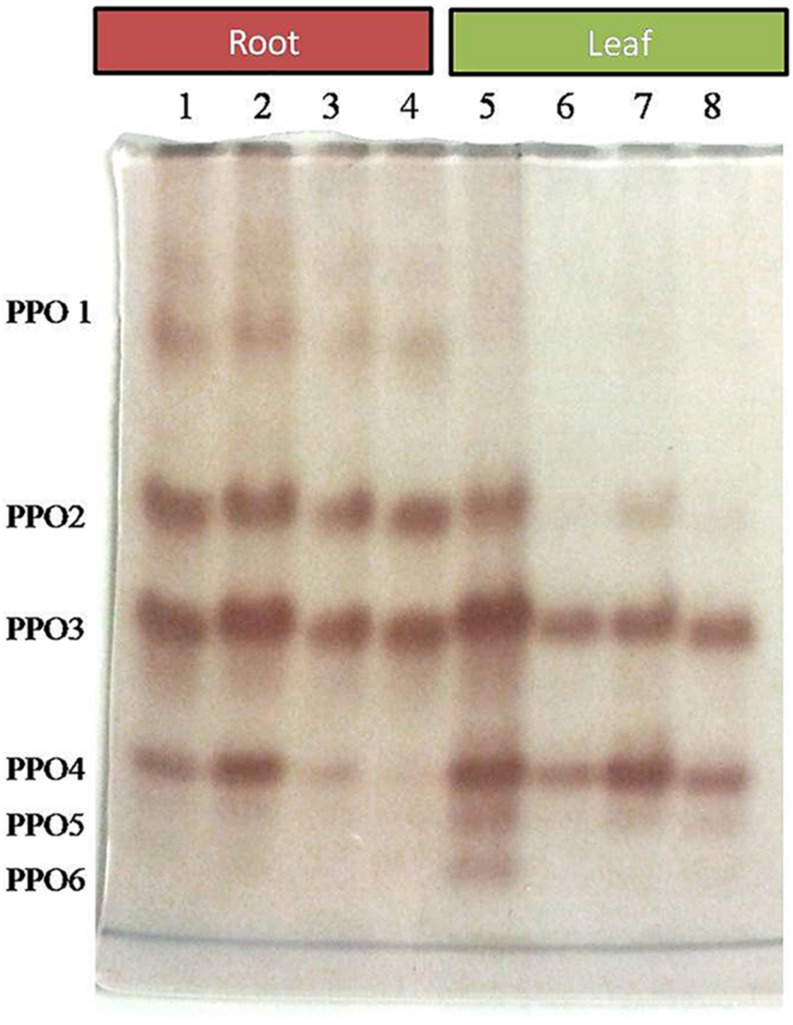
Zymography of PPO isoforms expressed in root and leaves upon bacterial (BHU-AV3) inoculation under salt stress. Lanes 1 and 5 – salt treatment; Lanes 2 and 6 – bacterial (BHU-AV3) inoculation + salt; Lanes 3 and 7 – bacterial inoculation; Lanes 5 and 8 – Control. PPO 1–5 represents number of isoforms expressed.

**FIGURE 9:**
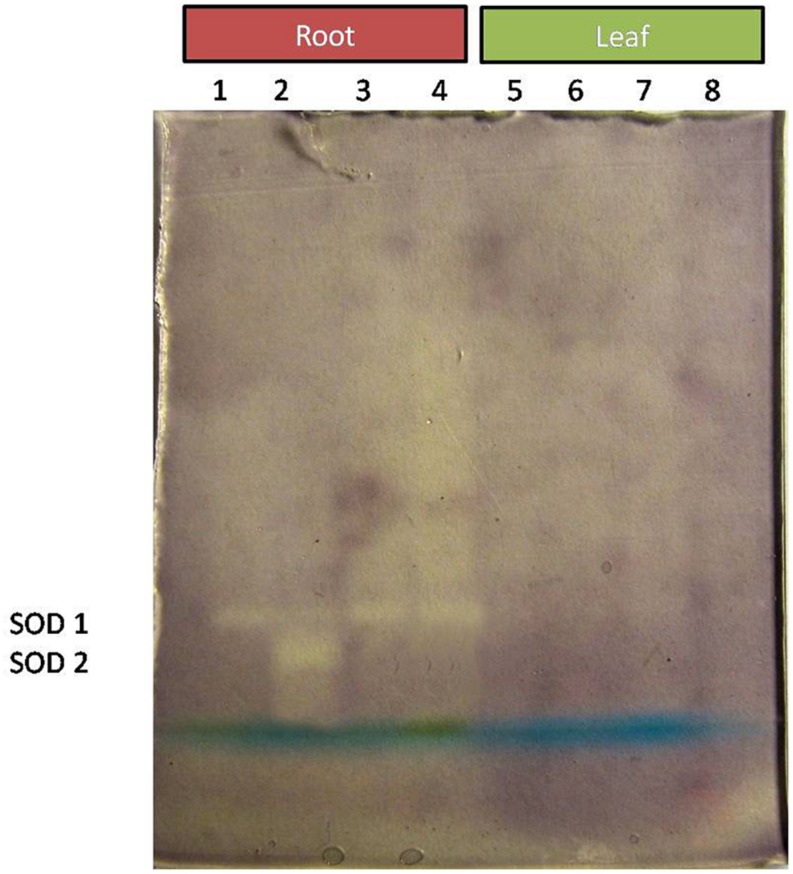
Zymography of SOD isoforms expressed in root and leaves upon bacterial (BHU-AV3) inoculation under salt stress. Lanes 1 and 5 – salt treatment; Lanes 2 and 6 – bacterial (BHU-AV3) inoculation + salt; Lanes 3 and 7 – bacterial inoculation; Lanes 5 and 8 – Control. SOD 1–5 represents number of isoforms expressed.

Comparative study of antioxidant enzyme activities was performed in root and leaf tissues with different treatments. Salt stress generally stimulates the antioxidant system throughout the plant. BHU-AV3 strain-inoculated roots (T4) showed higher POD (50%), SOD (29%), and PPO (16%) as compared to T2-treated plant roots. In leaf tissue, un-inoculated plants (T2) displayed significant increases in all enzymatic activities compared to T4 plants. Under non-salt conditions, both treatments (T1 and T3) maintained the same level of POD, SOD, and PPO activities (Figures 4–6). Further, the impact of salt stress on isozyme profiles was also explored. Native PAGE coupled with activity localization showed multiple isoforms of POD, PPO, and SOD in roots, while in leaves, faint bands were observed. Examination of POD isozyme profiles in the roots revealed five isoforms (POD1-5), and the activity of POD3, POD4, and POD5 was higher in T4 plants compared to T2 plants. In leaves, only one isoform, POD5, was detected in all treatments and exhibited higher expression in T4 plants (Figure 7). In the case of PPO activity, a total of six isoforms (PPO1-6) were expressed. Of these, PPO1 was expressed in root tissue only, whereas PPO5 and PPO6 were expressed only in leaves. In roots, the expressions of PPO2, PPO3, and PPO4 isozymes were found to be highest in T4 treatment compared to other treatments. In leaves, PPO2–PPO6 isoforms were highly expressed in the T4 treatment compared to the others (Figure 8). As shown in Figure 9, only two prominent SOD bands were detected in roots, while no band was found in leaves. The expression of SOD1 was higher in T4 plants compared to other treatments, while the SOD2 isoform was expressed only in T4 plant root.

### Effect of BHU-AV3 on *in situ* ROS Detection and the Effect of ROS on Plant Cells

**FIGURE 10:**
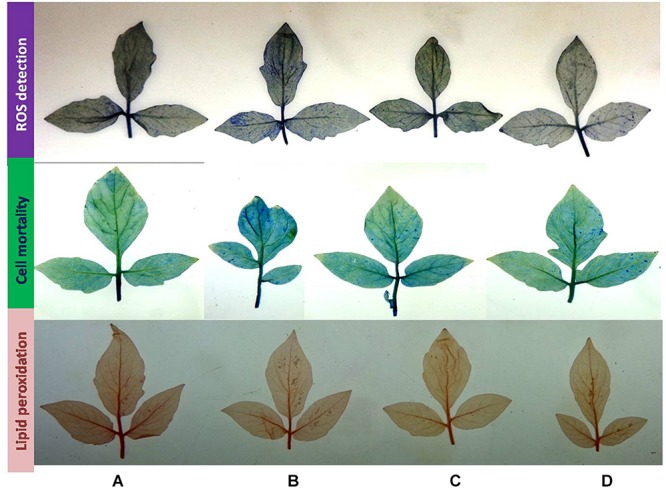
Histochemical analysis of cell death, ROS production, and lipid peroxidation in tomato plant leaves upon bacterial (BHU-AV3) inoculation under salt stress. ROS detection – blue spots show production of superoxide radicals; cell mortality – light blue spots show cell mortality; lipid peroxidation – red spots show lipid peroxidation. **(A)** Control; **(B)** salt (NaCl); **(C)** bacterial (BHU-AV3) inoculation; **(D)** bacterial inoculation + salt.

Superoxides were detected as bluish spots due to formazan formation on the leaf surface. In the presence of salt, leaves had higher staining, indicative of the production of ROS; however, plants inoculated with the strain BHU-AV3 (T4) showed lighter staining than un-inoculated plants (T2). In cell death analysis, un-inoculated plant leaves had more area of necrotic lesions in indigo blue spots compared to inoculated plants under salt stress. Lipid peroxidation was estimated through detection of malondialdehyde contents as pink spots on the leaf surface. T2-treated plant leaves exhibited a higher number of pinkish spots compared to T4-treated plants (Figure 10). These results suggest that strain BHU-AV3 reduced ROS content in salt-stressed tomato plants.

### Effect of BHU-AV3 on Root Protein Profiling in Response to Salt Stress

The differential expression of proteins produced in roots upon inoculation with BHU-AV3 under salt stress was investigated using a non-targeted approach. A total of 11 different protein bands were detected in tomato plant roots. The five highly expressed proteins in the T4 treatment were identified by MALDI-TOF/MS. Proteins were identified based on a high MASCOT score and peptide match (Table 4). The proteins expressed in T4 treatments were enolase, involved in the glycolytic pathway, ATP synthase, associated with energy metabolism, thiamine biosynthesis protein, elongation factor 1 alpha (EF1-alpha), involved in protein biosynthesis during the translation process, and catalase, associated with the ROS-scavenging process under stress conditions.

**TABLE 4:** Differentially expressed proteins in tomato plant roots upon bacterial inoculation (BHU-AV3) under salt stress conditions.

**Band number**	UniProtKB* **Accession**	Homologous protein**	**mW**^#^ **(Da)**	**pI**^$^	**PLGS**^$$^ **Score**	**Coverage**^¥^ **(%)**	**Matched**^£^ **Peptides**	**Molecular function**^¥¥^	**Biological**^£^ **function**
1	B9TU32	Thiamine biosynthesis protein ThiC variant L1 OS *Solanum lycopersicum* GN thiC PE 3 SV 1	72573	6.0337	836.6132	32.9231	9	ADP-ribose pyrophosphohydrolase activity	Response to vitamin B1
2	P30264	Catalase isozyme 1 OS *Solanum lycopersicum* GN CAT1 PE 2 SV 1	56470	6.5764	1237.124	38.2114	24	Catalase activity	Hydrogen peroxide catabolic process
3	Q2MI93	ATP synthase subunit beta chloroplastic OS *Solanum lycopersicum* GN atpB PE 3 SV 1	53433	5.0967	651.9818	28.1124	8	ATP binding	ATP synthesis coupled proton transport
4	P17786	Elongation factor 1 alpha OS *Solanum lycopersicum* PE 2 SV 1	49256	9.4614	651.9798	30.3571	14	GTPase activity	Translation
5	P26300	Enolase OS *Solanum lycopersicum* GN PGH1 PE 2 SV 1	47768	5.5746	1519.975	32.4324	12	Phosphopyruvate hydratase activity	Glycolytic process

**^∗^Accession number according to the UniProt database. ^∗∗^Homologous protein identified in the UniProt database. ^#^mW, molecular weight. ^$^pI, isoelectric point. ^$$^Ion score of identified protein using UniProt database.***^¥^***Sequence coverage; proteins with <25% sequence coverage were excluded from the result.***^£^***Proteins with more than three matched peptides were considered.*^¥¥^ Molecular Function, category using molecular functional classification by UniProt database. ^£^^£^Biological Function, category using biological functional classification by UniProt database.*

### Correlation of Protein Data With Gene Expression Analysis

**FIGURE 11:**
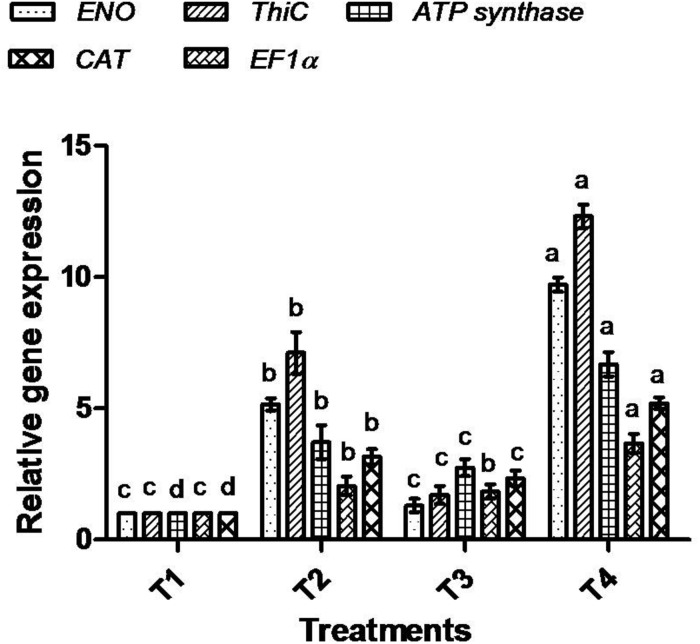
Effect of bacterial (BHU-AV3) inoculation on expression of selected proteins’ mRNA in roots under salt stress. T1 – control, T2 – salt (NaCl), T3 – bacterial (BHU-AV3) inoculation, and T4 – bacterial inoculation + salt. Values represent the mean ± SD, *n* = 3. Different letters on each bar indicate significant differences (*P* = 0.05) after DMRT test.

To correlate the protein data, gene expression analysis through qRT-PCR was performed for all five selected proteins. The mRNA expression of selected proteins was up-regulated in bacterially inoculated plant roots under salt stress (T4) in a similar way as determined by the 1-D PAGE analysis. In T2 treatment, plants exhibited less expression of all the tested mRNA genes compared to T4 plant roots (Figure 11).

## Discussion

While salt-tolerant PGPRs have received scientific attention due to their application in the reclamation of saline land, the molecular mechanism underpinning PGPR-mediated salt stress alleviation in plants has not yet been investigated systematically. Understanding of the molecular mechanisms involved is essential for developing salt-tolerant crop varieties (Chinnusamy et al., 2005; Vaishnav et al., 2014; Qin et al., 2016). In the present study, experiments were conducted to test the response elicited in tomato plants by inoculation with salt-tolerant BHU-AV3 under salt stress. The challenge by 200 mM NaCl resulted in reduced growth and biomass, imbalance of ions, decreased water content, and production of reactive oxygen species (ROS) in tomato plants. However, tomato plants inoculated with BHU-AV3 exhibited less senescence under 200 mM NaCl stress, positively correlated with the maintenance of ion balance, chlorophyll content, relative water content, and a low ROS level in plant cells.

Phylogenetic analysis of BHU-AV3 isolate using 16S rDNA sequencing revealed a similarity of the isolate with *Sphingobacterium* sp. Further, BHU-AV3 exhibited plant growth-promoting abilities that indicate the potentiality that the BHU-AV3 isolate would promote plant growth under nutrient-limited conditions. *Sphingobacterium* spp. are reported to have beneficial PGP traits, with plant growth-promotion abilities under different stress conditions (Marques et al., 2010; Ahmed et al., 2014; Cardinale et al., 2015; Rolli et al., 2015). In addition, the isolate BHU-AV3 was also found to tolerate salt stress up to 4% NaCl concentration. *Sphingobacterium* spp. are also reported to participate in soil remediation processes (Lodewyckx et al., 2002; Miliute et al., 2015).

BHU-AV3-inoculated plants had a higher biomass content under salt stress compared to un-inoculated plants, which is probably due to IAA production and nutrient solubilization activity of the BHU-AV3 strain in soil. Several other findings are also available on PGPR producing IAA and nutrient solubilization activities that helped increase plant growth and biomass accumulation in plants as an adaptive response to salt stress (Ali et al., 2014; Qin et al., 2014; Hahm et al., 2017; Pandey and Gupta, 2019). In the present work, a comparative study was performed in root and leaf tissues to evaluate the differences in physiological responses to salt stress upon inoculation with BHU-AV3. Interestingly, there were obvious differences in ion, antioxidant enzyme, and free proline contents in the leaves and roots of salt-stressed plants. A high salt concentration outside the root is known to cause ion imbalances in plants (Flowers and Yeo, 1986). Studies have reported a reduced level of internal K^+^ at high external NaCl concentrations (Horie et al., 2011; Wakeel, 2013). Due to this, an increased ratio of Na^+^/K^+^ was observed, which reduced plant growth and caused ionic toxicity (Hariadi et al., 2010; Abdelaziz et al., 2019). Our study revealed that salt stress induced a significant increase in Na^+^ content and Na^+^/K^+^ compared to the non-salt stress condition. It was observed that BHU-AV3-inoculated plant roots exhibited lower accumulations of Na^+^ and Na^+^/K^+^ compared to un-inoculated plants under salt stress. In addition, the accumulation of Na^+^ in leaves was less in bacterially inoculated plants compared to un-inoculated plant leaves. Several reports are available on PGPR mediation of salt tolerance in plants by reducing the transport of Na^+^ from the roots to leaves (Yasar et al., 2006; Zhang et al., 2013; Win et al., 2018; Romero-Munar et al., 2019). ROS are induced during salt exposure and lead to oxidative stress in plant cells (Miller et al., 2010; Sharma et al., 2012). Fortunately, plants have antioxidants to scavenge these enhanced ROS. In the present study, POD, PPO, and SOD enzyme activities were evaluated in tomato plants as a salt defense response, and augmented expression with a high number of isoforms of these enzymes was found in BHU-AV3-inoculated plant root. Four POD isoforms, two SOD isoforms, and four PPO isoforms with different molecular weights were significantly up-regulated in tomato plant roots inoculated with BHU-AV3 compared to un-inoculated plants under salt stress. The expression of multiple isoforms of antioxidant enzymes is involved in reducing the content of ROS and preventing the cell-damage (Kim et al., 2005; Zhang et al., 2013; Vighi et al., 2017; Arora and Bhatla, 2017; Sukweenadhi et al., 2018). In one study, new POD and SOD isoforms were expressed in response to salt stress tolerance in potato plants (Rahnama and Ebrahimzadeh, 2005). BHU-AV3-inoculated plant leaves had lower expression of antioxidant enzyme activities and fewer number of their isoforms compared to un-inoculated plant leaves under salt stress.

The roots of BHU-AV3-inoculated plants accumulated higher proline content compared to non-inoculated plant roots under salt stress, suggesting the role of higher proline in the maintenance of osmotic balance inside the root (Claussen, 2005; Zhu et al., 2019). In addition, increased proline content protects membrane proteins and enzymes from oxidative burst (Szabados and Savouré, 2010). Several studies have confirmed the ability of microbes to mitigate the effects of oxidative bursts by increasing the activity of osmolyte contents and antioxidant enzymes (Khanna et al., 2019; Rajput et al., 2019; Vaishnav et al., 2019; Zahir et al., 2019). Interestingly, BHU-AV3-inoculated plant leaves exhibited less proline content compared to un-inoculated plants. The differences in antioxidant enzyme activities and free proline content between leaves and roots might be due to different metabolisms and functions of tissues (Cavalcanti et al., 2007). A few studies have reported on variability in the salt stress response in separate plant tissues upon bacterial inoculation (Cardinale et al., 2015; He et al., 2018). In another explanation, lower accumulation of proline and antioxidant enzymes indicates that plants are less affected by salt stress (Kohler et al., 2009; Zhang et al., 2013; Latef and Chaoxing, 2014). Negrão et al. (2017) described that root tissues are more prone to salt stress compared to shoot and leaf, as they are directly in contact with soil. Our results suggest that BHU-AV3 reduced osmotic and oxidative stress in plants by inducing proline content and antioxidant enzyme activities in roots, which are basically exposed to the salt stress. This hypothesis is also supported by results of *in situ* detection of ROS, lipid peroxidation, and cell death. BHU-AV3-inoculated plant leaves had less accumulation of ROS content and lower lipid peroxidation and cell death under salt stress compared to un-inoculated plant leaves.

In this study, one of our major focuses was on induced salt stress-responsive proteins in tomato plant roots upon inoculation with BHU-AV3. After a non-targeted protein study, we identified five proteins that were highly expressed under salt stress conditions, namely enolase, ATP synthase, thiamine biosynthesis protein, elongation factor 1 alpha (EF1-alpha), and catalase. The protein expression was further correlated by target-based gene expression analysis of the selected proteins. The gene expression analysis confirmed the up-regulation of all of the tested genes expression in BHU-AV3-inoculated plant roots. The consistency between the protein expression level and transcription level of the five selected protein genes manifests that the expression of these proteins may be controlled at the transcriptional level during bacterial interaction with plants under salt stress (Zhang et al., 2015; Cao et al., 2017).

Induction of energy metabolism under salt stress can be addressed by an increase in the abundance of ATP synthase and enolase proteins in tomato plants upon bacterial inoculation, while un-inoculated plants had lower expression of the same proteins. In a similar way, overexpression of ATP synthase in roots led to greater tolerance to salt stress in plants (Zhang et al., 2006; Li et al., 2011; Agrawal et al., 2016; Cao et al., 2017).

In addition, enolase is an important enzyme of the glycolysis pathway. Studies are available showing that increased ENO gene expression under salt stress generates more energy to cope with stress (Yan et al., 2005; Barkla et al., 2009; Zhang et al., 2018). The glycolysis pathway is the best way to generate energy quickly under normal (non-stress) conditions (Kürsteiner et al., 2003). In the present study, enhancement in the synthesis of ATP synthase and enolase upon bacterial inoculation suggests that these proteins may play important roles in maintaining the energy state and protecting plants against salt stress conditions.

An increase in expression of thiamine protein was observed in BHU-AV3-inoculated plant roots under salt stress. The thiamine synthesis protein supplies thiamine pyrophosphate (TPP) for several metabolic processes in plants (Goyer, 2010). In addition, thiamine is also involved in plant adaptations to different abiotic stresses (Tunc-Ozdemir et al., 2009; Rapala-Kozik et al., 2012). In one study, an exogenous application of thiamine was found to induce salinity tolerance in plants (Sayed and Gadallah, 2002).

EF1-alpha protein is involved in the initiation and elongation stage of mRNA translation and protein synthesis. The higher expression of EF1-alpha protein in BHU-AV3-inoculated plant roots suggests its participation in higher protein synthesis to protect the plant cells against salt toxicity, as previously explained by Shin et al. (2009) and Fercha et al. (2013). In addition, EF1-alpha proteins were also reported to perform a chaperone function by interacting with unfolded proteins, thereby protecting them from aggregation under stress conditions (Ristic et al., 2007; Bukovnik et al., 2009).

Catalase (CAT) is known for its antioxidant nature. It converts hydrogen peroxide (H_2_O_2_) into water and oxygen. It is highly specific for H_2_O_2_ and does not require a reductant in activity. A significant induction of CAT removed the ROS produced during salt stress in BHU-AV3-inoculated plants. Our results are in conformity with other findings that report enhanced activities of CAT enzymes in PGPR-inoculated plants under oxidative stress (Chen et al., 2016; Mesa-Marín et al., 2018; Afridi et al., 2019).

## Conclusion

The current report extends our understanding of the salt tolerance mechanisms in tomato plants following inoculation with a salt-tolerant PGPR strain, BHU-AV3. Our findings clearly demonstrated that inoculation with BHU-AV3 increased salt tolerance in tomato plants and that the roots were physiologically more responsive to alleviating salt stress. The tomato plant roots showed more severe changes in accumulating Na^+^, proline, and antioxidant enzymatic activities compared to the leaves under salt stress with BHU-AV3 inoculation. Enhanced activities of these parameters in roots resulted in a decrease in oxidative stress in tomato plants, as measured in leaves with respect to ROS content, MDA content, and cell death assay. The protein study revealed that carbohydrate and energy metabolism, antioxidative enzymes, and translation-related proteins were up-regulated in BHU-AV3-inoculated plant roots in response to salt stress. These proteins may work cooperatively to enhance salt tolerance and enable them to survive under severe stress. Insights gained from such research increases our understanding of plant–microbe interactions and could aid in engineering plants with improved salt tolerance. In addition, such salt-tolerant PGPRs boost the potential to decrease the use of agrochemicals on cultivated land and perhaps enhance crop productivity on saline soils around the world.

## Data Availability Statement

The datasets generated for this study can be found in the NCBI GenBank, Accession number MK588751.

## Author Contributions

AV designed and conceived of the research and drafted the manuscript. JS and PS helped in most of the experimental work. RR helped in the compilation of data and interpretation of the results. HS and BS coordinated the work and edited the manuscript.

## Conflict of Interest

The authors declare that the research was conducted in the absence of any commercial or financial relationships that could be construed as a potential conflict of interest.
